# Emerging microRNA Therapeutic Approaches for Cystic Fibrosis

**DOI:** 10.3389/fphar.2018.01113

**Published:** 2018-10-08

**Authors:** Pauline Bardin, Florence Sonneville, Harriet Corvol, Olivier Tabary

**Affiliations:** ^1^INSERM UMR-S938, Centre de Recherche Saint Antoine, Faculté des Sciences, Sorbonne Université, Paris, France; ^2^Paediatric Respiratory Department, Hôpital Trousseau, Assistance Publique – Hôpitaux de Paris, Paris, France

**Keywords:** cystic fibrosis, miRNA, therapy, oligonucleotides, CFTR (Cystic Fibrosis Transmembrane conductance Regulator), ANO 1 channel

## Abstract

Cystic fibrosis (CF) is caused by mutations in the cystic fibrosis transmembrane conductance regulator *(CFTR)* gene and remains the most common life-shortening diseases affecting the exocrine organs. The absence of this channel results in an imbalance of ion concentrations across the cell membrane and results in more abnormal secretion and mucus plugging in the gastrointestinal tract and in the lungs of CF patients. The direct introduction of fully functional CFTR by gene therapy has long been pursued as a therapeutical option to restore CFTR function independent of the specific CFTR mutation, but the different clinical trials failed to propose persuasive evidence of this strategy. The last ten years has led to the development of new pharmacotherapies which can activate CFTR function in a mutation-specific manner. Although approximately 2,000 different disease-associated mutations have been identified, a single codon deletion, F508del, is by far the most common and is present on at least one allele in approximately 70% of the patients in CF populations. This strategy is limited by chemistry, the knowledge on CFTR and the heterogenicity of the patients. New research efforts in CF aim to develop other therapeutical approaches to combine different strategies. Targeting RNA appears as a new and an important opportunity to modulate dysregulated biological processes. Abnormal miRNA activity has been linked to numerous diseases, and over the last decade, the critical role of miRNA in regulating biological processes has fostered interest in how miRNA binds to and interacts explicitly with the target protein. Herein, this review describes the different strategies to identify dysregulated miRNA opens up a new concept and new opportunities to correct CFTR deficiency. This review describes therapeutic applications of antisense techniques currently under investigation in CF.

## Introduction

Cystic fibrosis (CF) is the most common lethal genetic disease in the Caucasian population. Since the gene responsible for the disease was identified in 1989 (Riordan et al., 1989), numerous discoveries have led to an improvement in patients’ longevity and quality of life. It is a complex disease, and many advances are still needed to understand the mechanisms of the pathology, to cure patients.

Cystic fibrosis affects about 70,000 people worldwide (in the homozygous or heterozygous composite state). It is induced by the recessive mutation of the gene that encodes a chloride channel called CFTR for Cystic Fibrosis Transmembrane conductance Regulator (Riordan et al., 1989). The *CFTR* gene is located on the long arm of chromosome 7 (7q31) and encoded for the CFTR protein composed of 1,480 amino acids and belongs to the family of ABC transporters (ATP-binding cassette transporters) whose central role is the active transport of various substrates, such as amino acids, peptides, proteins, and ions through the plasma membrane. The CFTR protein participates directly in the regulation of the transport of chloride ions at the cell membrane, as well as in the transport of bicarbonate ions, which are involved in the regulation of the pH of the airway surface liquid covering the pulmonary epithelium (Jun et al., 2016).

From a pathophysiological point of view, mutations cause alterations in the tissues or organs integrating the exocrine glands, such as the skin, pancreas, lungs, intestines, and reproductive systems. Currently, the leading cause of morbidity and mortality is related to damage to the lungs. They are characterized by an accumulation of thick, viscous mucus, recurrent infections, and chronic inflammation, causing an impairment of mucociliary clearance. Inflammation could appear before any infection; the secondary infection would only exacerbate the uncontrollable inflammation (Jacquot et al., 2008). In the long term, these various symptoms lead to the deterioration of the pulmonary epithelium with, as a result, repair mechanisms being activated at the root of the appearance of areas of epithelial rearrangement. Alongside the peeling and remodeling of the epithelium, subepithelial fibrosis develops, leading to impaired respiratory function.

To date, more than 2,000 mutations of the CFTR gene have been identified, the most common (found in nearly 70% of patients) being the F508del mutation of the protein (deletion of a phenylalanine at position 508). These mutations are divided into six classes according to their degree of severity in CF disease and the mechanism that disrupts CFTR function and induced a tremendous phenotypic variability of CF patients (Corvol et al., 2016). CFTR mutation classes were created according to their consequences on CFTR function mutations interfering with protein synthesis (class I), mutations affecting protein maturation (class II), mutations altering channel regulation (class III), mutations affecting chloride conductance (class IV), mutations reducing the level of normally functioning CFTR at the apical membrane (class V), and mutations decreasing the stability of CFTR present at the plasma membrane (class VI) (Fanen et al., 2014). The high number of these mutations and their effects on the functionality of the protein make an unique therapeutic approach complicated^1^.

## Cystic Fibrosis and Therapeutic Approaches

At the moment, there is no cure for CF. The increase in life expectancy observed in recent years is in fact mainly due to better management of the disease with symptomatic treatments (physical therapy, mucolytic agents, anti-inflammatories, and antibiotics). New pharmacological therapeutic approaches (Vertex Pharmaceuticals) have been proposed to patients for less than a decade. They help to restore the defective protein to the cell membrane (correctors) and/or to potentiate its activity (potentiators). The first small molecule that has demonstrated efficacy is ivacaftor. Preclinical and different clinical studies have shown that ivacaftor corrects CFTR-mediated chloride transport in most class III mutations, class IV mutations, and some other residual function mutations (Bessonova et al., 2018). A subsequent series of clinical trials have shown that ivacaftor has a high level of efficacy in class III mutations, particularly in patients with G551D (Gly551Asp) mutation even in very young children (Rosenfeld et al., 2018). In all clinical studies, compared with placebo, treatment with ivacaftor improved lung function (FEV1) by around 10%, reduced sweat chloride concentration by around 60 mmol/L, improved quality of life, and reduced the frequency of pulmonary exacerbations. The second small molecule strategy has been to target patients who are homozygous for the F508del mutation with a combination of a corrector drug to restore trafficking of CFTR and a potentiator to make it functional even if the activity of the channel remained low. The different clinical trials have demonstrated an improvement of 2.8 to 3.3% in FEV1 read out, that reflect a small effect for a high cost that induces a debate in the different national institute for health (Gulland, 2016). Due to the heterogeneity of the mutations that affect the *CFTR* gene from patient to patient (Veit et al., 2016), these treatments are not applicable to everyone and therefore have a highly variable benefit, depending on the class mutation (Gulland, 2016). Unfortunately, at present, there is no treatment that would treat all patients and in particular patients with a class I mutation. These drug classes, however, are a frame of reference in the field, and new molecules based on the same strategy are still under development. However, other strategies are proposed at the same time. They improve mucociliary clearance, decrease inflammation or fight infection^2^. One of the new therapeutic strategies proposed in the literature is based on the stimulation of an alternative pathway to that involving CFTR for the transfer of chloride ions, such as that induced by the chloride channel ANO1 (Anoctamin-1, also called TMEM16A [transmembrane protein 16A]) (Caputo et al., 2008; Schroeder et al., 2008; Yang et al., 2008; Sonneville et al., 2017), which could also participate in the activation of CFTR in the cell membrane (Benedetto et al., 2017). Another proposed emerging approach is based on modulating the expression of the microRNAs that regulate target RNAs such as CFTR.

## miRNAS: CFTR and Other Associated Channel Proteins

**FIGURE 1:**
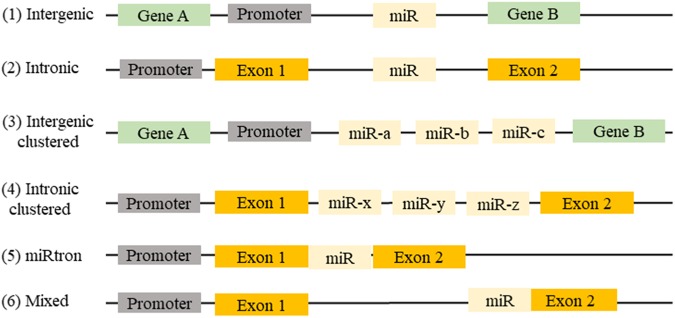
Location of miRNAs gene in the genome: (1) miRNAs can be found between two genes (intergenic) or (2) in a gene (intronic). (3) They may be present in a single miRNA gene or (4) in a cluster of miRNA genes. (5) Sometimes intronic miRNAs may exist between two exons (miRtron) or (6) overlapping an exon and an intron of non-coding genes (mixed).

MicroRNAs (miRNAs) are defined as small, non-coding, 21–23 nucleotide endogenous RNAs able of suppressing the expression of their target genes. miRNAs can be classified on their genomic location and gene structure, and in the miRBase database, more than 28,000 miRNAs are referenced (Kozomara and Griffiths-Jones, 2014). Almost half of the known miRNA genes are located in intergenic regions. Intronic miRNA, are located in the introns of annotated genes, including both coding and non-coding genes. Intergenic and intronic miRNAs gene may exist as a single gene or a cluster of genes under the control of their own promoters. Mixed miRNA are found overlapping across an exon and an intron of non-coding genes (Wang and Cai, 2017; **Figure 1**).

**FIGURE 2:**
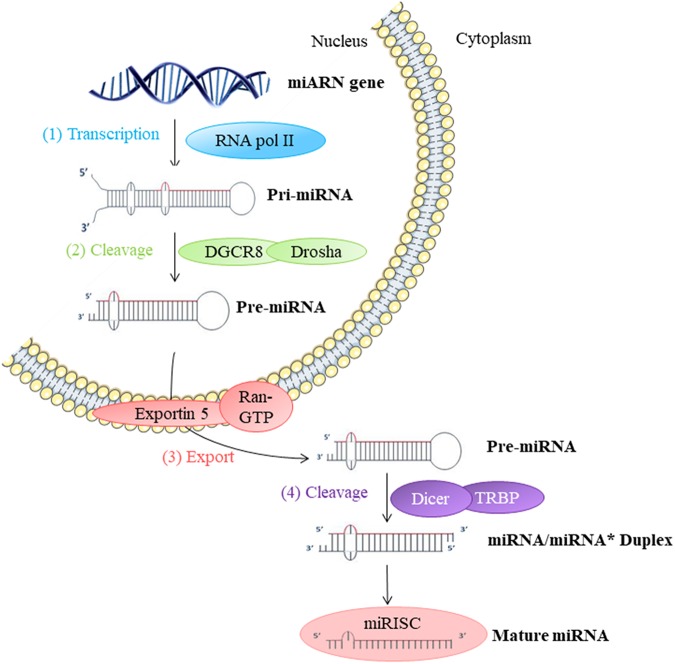
Biosynthesis of miRNAs: (1) The biosynthesis begins in the nucleus by transcription of miRNA genes by RNA polymerase II (Pol II). (2) Long transcripts, pri-miRNA, are cleaved by Drosha and DGCR8 protein creating pre-miRNA with hairpin structure. (3) Exportin 5 transfers pre-miRNA into the cytoplasm. (4) Pre-miRNA is cleaved by Dicer into miRNA duplex in mature single-strand miRNA form. This miRNA mature form is incorporated into a miRISC ribonucleoprotein complex. This complex can then act directly on the mRNA in the cell where it is synthesized or out of the cell.

**FIGURE 3:**
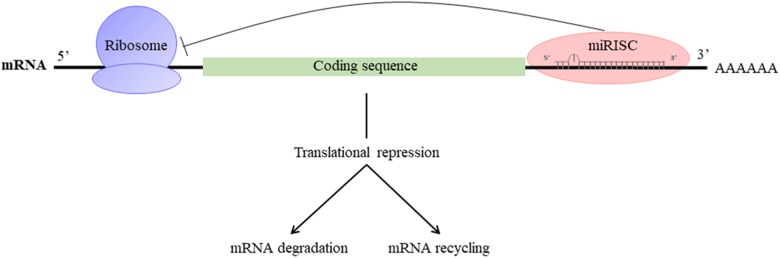
Mechanism of action of miRNAs: miRISC complex attenuates mRNA translation and leads to the destabilization of mRNA by deadenylation and/or inhibition of translation.

They participate in the regulation of many cellular processes, and the impairment of their expression may be associated with some pathologies (Foster et al., 2013). miRNAs are produced by transcription and maturation of their mRNA, ultimately with the release of a mature miRNA that will be incorporated into a ribonucleoprotein complex called miRISC (miRNA-induced silencing complex; **Figure 2**). Traditionally, the regulation exerted by miRNAs on their target gene is specific, through the pairing of the mature miRNA with the 3′-UTR sequence of the target mRNA, called miRNA response element (MRE) (**Figure 3**). The activity of the miRNAs depends on a long sequence of only 6 nucleotides (the “seed” sequence). An MRE motif can, therefore, be found on different mRNAs, and a mRNA can contain a repetition of the same MRE motif or other motifs. miRNAs play a dominant role in the complex multiple-gene expression regulation networks (Sonneville et al., 2015). When talking about a therapeutic approach, it is essential to take into account the emergence of isomiR, the 3′ and 5′ sequence variants of canonical miRNAs, as well as the wrong annotations that can be found in the database. The data collected by sequencing also need to be systematically verified, as described previously for isomiR-34/449, involved in ciliogenesis (Mercey et al., 2017). Today, it is estimated that more than 60% of the genes encoding a protein are regulated by miRNAs (Friedman et al., 2009). Modulating the activity of a single miRNA as a result of the inhibition of its function or overexpression can thereby have a significant biological impact.

Many studies have focused on the potentially regulatory miRNAs of *CFTR* expression in CF. Gillen et al. (2011) were the first to identify miRNAs related to CFTR activity, several of them directly regulated its expression by binding to the 3′-UTR end of its mRNA. The regulation of *CFTR* mRNA expression differs according to the cell type being studied: it is, in fact, tissue-specific, and time-dependent, which makes the development of miRNA-targeting therapies meant to restore the function of CFTR complicated (**Table 1**). Thus, Viart et al. (2015) showed that *CFTR* regulation was different before and after birth, confirming previous results CFTR channel is strongly expressed but it falls after birth at the pulmonary level (Marcorelles et al., 2007). In fact, miR-101 negatively regulates *CFTR* expression in adult lung cell lines, but it has no effect on fetal bronchial epithelial cells. It, therefore, seems to play a crucial role, which evolves over time (Viart et al., 2015). The combination of this miRNA with miR-494 makes it possible to remarkably suppress the expression of CFTR in human renal embryos. *In silico* analyzes have shown that these two miRNAs can target genes concomitantly and thus influence the severity of CF patients’ pathology (Megiorni et al., 2011). The direct link between miRNA and *CFTR* has subsequently been confirmed by other teams who have shown that miR-145, miR-223, and miR-494, alone or together synergistically, directly regulate *CFTR* expression. This is also the case for miR-509-3p and miR-494, which induce stronger repression of its expression (Oglesby et al., 2013; Ramachandran et al., 2013). In addition to being able to interact directly with *CFTR*, some miRNAs can also act on intermediate actors of its biosynthesis, such as the transcription factor *SIN3A* (SIN3 transcription regulator family member A), repressed by miR-138 (Ramachandran et al., 2012; **Table 1**). They showed that the overexpression of miR-138 contributes to increase the expression and the number of CFTR channels on the cell surface of secretory epithelia but also the activity of the channel, in healthy subjects and CF patient with F508del mutations even if the mutated channel is less active than the wild-type. All these results were mainly performed on mutated F508del CFTR protein and should be also performed on other class mutations. The implication of miRNAs in CFTR expression is also controlled by the highly variable, and compelling information on phenotypic variability and lack of genotype-phenotype correlation among patients with the same mutation in the CFTR gene. Thus, three polymorphisms have been identified in the miR-99b/let-7e/miR-125a cluster that modulates the expression of these miRNAs and may be associated with patient phenotypes (Endale Ahanda et al., 2015). In addition, polymorphisms present in the 3′-UTR region of CFTR mediate the binding of miR-509-3p and cause a decrease in CFTR expression (Amato et al., 2013).

**Table 1:** Table showing deregulated miRNAs targeting directly or indirectly CFTR in the context of cystic fibrosis.

Target	miRNA			Models		Reference		Year
CFTR mRNA	miR-494, miR-384, miR-376b, miR-1246, miR-145, miR-331-3p et miR-939			Caco-2		Gillen et al., 2011		2011
	miR-600, miR-494, miR-384, miR-1290, miR-1246, miR-145, miR-1827, miR-331-3p et miR-939			PANC-1				
	miR-600, miR-494, miR-607 et miR-384			16HBE14o-				
	miR-101, miR-494			HEK293		Megiorni et al., 2011		
	miR-101, miR-144			16HBE14o-		Hassan et al., 2012		2012
	miR-145, miR-223, miR-494			Bronchial brushing, 16HBE14o-, CFBE41o-, HEK293		Oglesby et al., 2013		2013
	miR-509-3p, miR-494			Differentiated primary cell cultures, Calu-3		Ramachandran et al., 2013		
	miR-145, miR-384, miR-101, miR-600			A549		Viart et al., 2015		2015
	miR-505, miR-943, miR-377, miR-384, miR-101, miR-600			Beas-2B				
	miR-600			HBEpiC				
	groupe miR-17-92			Human macrophages		Tazi et al., 2016		2016
	miR-145-5p			Calu-3		Fabbri et al., 2017		2017
	miR-200b			Calu-3, 16HBE14o-		Bartoszewska et al., 2017		
	miR-145			CF and non-CF differentiated primary cell cultures		Lutful Kabir et al., 2017		
CFTR mRNA through SIN3A mRNA	miR-138			Differentiated primary cell cultures, Calu-3, HEK293, HeLa		Ramachandran et al., 2012		2012

Expression of miRNAs can also regulate the expression of other channels or others proteins whose activity is modulated by CFTR (**Table 2**), such as the cotransporter NKCC1 (Na^+^-K^+^-2Cl^-^-cotransport protein) (Gillen et al., 2011) or the sodium channel ENaC (epithelial sodium channel) by miR-183, which is able to target the three subunit of ENaC (Kim et al., 2017). Recently, an increase in the expression of miR-9 was at the root of a decrease in expression of the chloride channel, ANO1, which modulated mucus hydration and chloride efflux activity (Sonneville et al., 2017).

**Table 2:** Table showing deregulated miRNAs direct/indirect targeting others targets in the context of cystic fibrosis.

miRNA	Target	Models		Reference	Year
miR-126	TOM1 Target of Myb1 Membrane Trafficking Protein	Bronchial brushing, 16HBE14o-, CFBE41o-, HEK293		Oglesby et al., 2010	2010
miR-155	SHIP1 (indirect: IL-8) SH-2 containing inositol 5′ polyphosphatase 1	IB-3, S9		Bhattacharyya et al., 2011	2011
miR-101, miR-1246, miR-494 et miR-384	SLC12A2 Solute Carrier family 12 Member 2	PANC-1		Gillen et al., 2011	
miR-449	Notch1 Notch homolog 1	Differentiated primary cell cultures		Marcet et al., 2011	
miR-146a	MUC5AC Mucin 5 AC	16HBE14o-		Zhong et al., 2011	
miR-145	SMAD3 Mothers Against Decapentaplegic homolog 3	Nasal epithelium cells, HEK293		Megiorni et al., 2013	2013
miR-31	IRF1 (indirect: Cathepsin 5) Interferon Regulatory Factor 1	Differentiated primary cell cultures		Weldon et al., 2014	2014
miR-93	IL-8 Interleukin 8	IB3-1, CuFi-1, NuLi-1		Fabbri et al., 2014	
miR-17	IL-8	Bronchial brushing, 16HBE14o-, CFBE41o-, HEK293		Oglesby et al., 2015	2015
miR-221	ATF6 Activating Transcription Factor 6				
miR-199a-5p	CAV1 Caveolin 1	Human and murine macrophages from lungs		Zhang et al., 2015	
miR-155	RPTOR Regulatory Associated Protein of mTOR complex	IB3-1, S9		Tsuchiya et al., 2016	2016
miR-199a-5p	TβRII TGF beta receptor II	Stellar hepatic cells		Chen et al., 2016	
miR-1343	TGF-β receptor	A549, 16HBE14o-, Caco-2		Stolzenburg et al., 2016	
miR-145	TGF-β Transforming growth factor beta	Primary cells from CF and non-CF patients		Lutful Kabir et al., 2017	
miR-183	SCNN1α,β,γ Sodium Channel Epithelial 1 alpha, beta, gamma subunit	CFBE41o-		Kim et al., 2017	2017
miR-9	ANO1 Anoctamin 1 (TMEM16A)	16HBE14o-, CFBE41o-		Sonneville et al., 2017	
miR-199a-3p	IKKβ Inhibitor of Kappa light polypeptide gene enhancer in beta cells, kinase beta	CFBE41o-		Bardin et al., 2018	2018

## miRNAs: Obstruction, Infection, and Inflammation

In recent years, studies showing the involvement of miRNAs in the regulation of other targets involved in various aspects of the pathology, such as inflammation, airway obstruction or infection, have opened up new ways of investigation. In fact, miRNAs are able to modulate the expression of genes involved in airway obstruction and the production of mucus, such as miR-146a modulating *MUC5AC*, which encodes one of the main pulmonary mucins implicated in the obstruction of the airway. The data highlight a negative feedback role for miR-146a in the control of MUC5AC production from airway epithelial cells stimulated by neutrophil elastase, which may be associated with the inactivation of MAP kinase and NF-κB signaling, the primary pathways implicated in CF airway inflammation (Zhong et al., 2011). The expression of miR-101 in the airways is therefore increased in the lungs of patients with CF, with an airway obstruction characterized by low levels of *CFTR* (Hassan et al., 2012) expression. miRNA expression is also influenced by external factors, like pathogenic bacteria, such as *Haemophilus influenza, Staphylococcus aureus*, or *Pseudomonas aeruginosa* (Oglesby et al., 2013; Ramachandran et al., 2013). The presence of pathogens in the light of CF airway surface epithelium contributes to modulate miR expression and consequently CFTR expression. In an infectious diseases context (e.g., in the presence of *P. aeruginosa*), miR-93 expression, which is decreased, is associated with stabilization of IL-8 mRNA, thereby contributing to the maintenance of the inflammatory state. Altogether, the different works highlighted some complexes regulation and needed further investigation (Fabbri et al., 2014).

In the airways, inflammatory processes controlled by NF-κB are also partially regulated by miRNAs. The miR-199a-3p expression in the airway, which is decreased in CF context, is associated with an NF-κB hyperactivation. In fact, miR-199a-3p directly regulates IKKβ, which acts on the NF-κB signaling pathway and therefore on IL-8 secretion, the main cytokine dysregulated in the airways of CF patients (Bardin et al., 2018). Moreover, miR-509-3p, miR-494, and miR-126 directly target NF-κB and have shown that CFTR expression and function decreases when NF-κB is functional (McKiernan et al., 2013; Ramachandran et al., 2013). The two miR-93 and miR-17 regulate the production of IL-8 in bronchial epithelial cells (Fabbri et al., 2014; Oglesby et al., 2015). Bhattacharyya et al. (2011) therefore showed that miR-155 expression was associated with CFTR activity. This miRNA can directly regulate SHIP1 (SH-2 containing inositol 5′ polyphosphatase 1) and indirectly alter the expression of IL-8 through activation of the signaling pathway involving PI3K/Akt (phosphatidylinositol-3 kinase/protein kinase B) and inhibition of the MAPKs (mitogen-activated protein kinases) (Bhattacharyya et al., 2011). The origin of the deregulation of miR-155 expression in CF cells is mediated by TTP (tristetraprolin) and KSRP (KH-type splicing regulatory protein) known to regulate miRNA biogenesis. The origin of this miRNAs dysregulation needs to be assessed. Some miRNAs may participate in the remodeling of the pulmonary epithelium and have a significant role in the development of the pathophysiology of the disease. Expression of miR-449 in the respiratory epithelium is essential to inducing direct inhibition of the Notch pathway and modulation of that involving the small GTPases, two events necessary for the production of motile cilia the beating of which allows mucus to be evacuated (Marcet et al., 2011; Chevalier et al., 2015). The remodeling of the pulmonary epithelium can also be caused by deregulation of TGF-β (transforming growth factor-beta) pathway, both in the expression of its receptors (TGF-βR1 and R2), and in signaling intermediates such as RPTOR (regulatory associated of mTOR complex 1) resulting in increased fibrosis and CTGF (connective tissue growth factor) or SMAD proteins, respectively through miR-1343, miR-155 and miR-145, thereby affecting fibrotic markers, cell migration and epithelial-mesenchymal transition (Stolzenburg et al., 2016; Tsuchiya et al., 2016; Fabbri et al., 2017). The deregulation of miR-31 in the airways of patients with CF contributes to pulmonary inflammation by increasing the activity of cathepsin 5, which causes antimicrobial proteins to deteriorate (Weldon et al., 2014). As for miR-221, it controls the transcription factor *ATF6* (activating transcription factor 6), which is involved in inflammation caused by endoplasmic reticulum stress (Oglesby et al., 2015). At last, miR-199a-5p regulates caveolin 1 (CAV1), a protein involved in the resolution of inflammation processes, targeting the PI3K/Akt/CAV1 axis (Zhang et al., 2015) and the TGF-β pathway (Lino Cardenas et al., 2013). Furthermore, it has been shown that Celecoxib, which modulates the Akt/miR-199a-5p/CAV1 axis, improves pulmonary hyper-inflammation caused by macrophages in patients.

## miRNAs: Therapeutic Strategies

**FIGURE 4:**
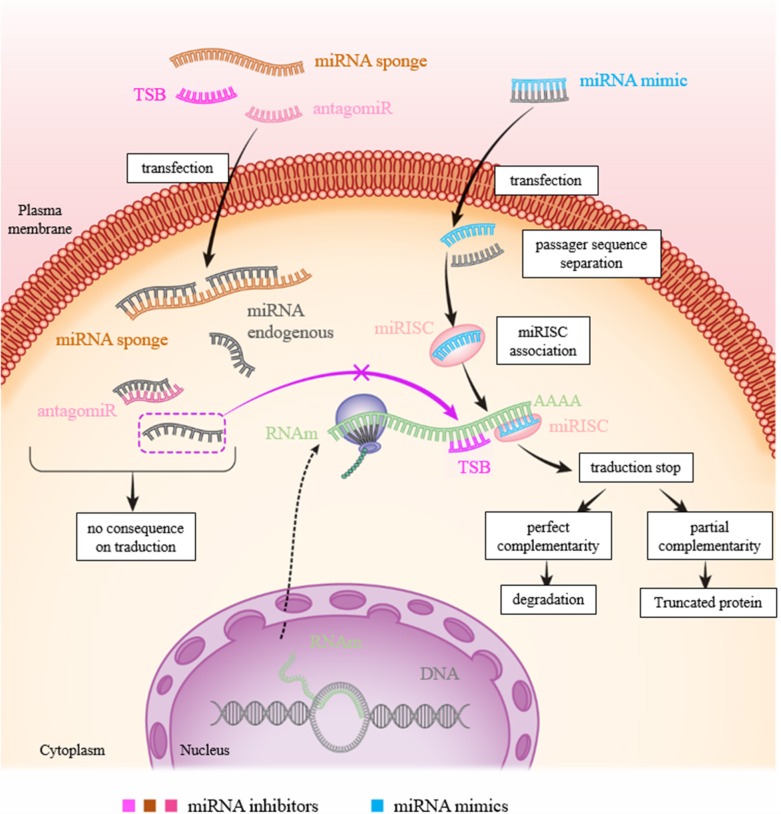
Figure showing the various approaches to inhibit or mimic a miRNA. In physiological condition: the miRNA will bind to the 3′-UTR of its target to inhibit translation resulting to RNA degradation and/or mRNA recycling. To inhibit a miR action, there are three possibilities: transfection of (1) miRNA inhibitor (antagomiR): the miRNA inhibitor binds to the latter by complementarity and prevents its action on its target; (2) TSB: the TSB specifically binds to the 3′-UTR of the target mRNA at the miRNA binding site, the miRNA can then no longer attach to it and exert its action; and (3) mRNA sponge: the miRNA is saturated by the miRNA sponge which contains many miRNA complementary sequences, the miRNA can then no longer attach itself to its target. To increase the expression of a miR, transfection of a mimic of this miR is sufficient to inhibit translation of mRNA.

MicroRNAs (miRNAs) are now considered as potential therapeutic targets. Two approaches have been followed for developing therapies based on their use: antagonists also called antisense oligonucleotides (ASOs) including inhibitors, miR sponges and target site blocker [TSB]) and miRNA mimics (**Figure 4**).

miRNA mimics are RNAs mimicking endogenous molecules and helping amplify their function. They are used to restore a loss of function. The purpose of this so-called “miRNA replacement therapy” approach is to reintroduce miRNAs whose expression is reduced in a pathological context. The proof of concept of this miRNA replacement therapy has been demonstrated with the use of miRNA tumor suppressors that stimulate anti-oncogenic signaling pathways and lead to the eradication of tumor cells (Wiggins et al., 2010). Let-7 and miR-34 mimics are currently in the clinical development phase to target a broad spectrum of tumors. The first molecule aimed at increasing the expression of miR-34 through the use of a mimic (MRX34) entered phase I clinical trials in 2013, as part of studies on multiple solid tumors (Bouchie, 2013). The main limitation of this approach is the difficulty formulating the mimic for its delivery to the target cells. One of the existing strategies to remedy, this is their coupling to nanoparticles. In the clinical trial using MRX34, the mimic is encapsulated in a liposome delivery system to facilitate its uptake by the target cells (Bouchie, 2013). An alternative approach uses delivery through miRNA expression vectors using adenovirus infection as previously described in a cancer therapy approach (Kota and Balasubramanian, 2010).

As for miRNA antagonists, they are used to inhibit endogenous miRNAs that show a-gain-of function in a pathological context. These therapies are similar to using siRNA (small interfering RNA), even through the regulatory systems involved are different. The miRNA antagonist or antagomiR (also called antimiR) binds to the mature miRNA targets with a powerful affinity. The duplex thus formed will then deteriorate. The main disadvantage of this type of therapy is that a miRNA can regulate the expression of several genes. Inhibition of a miRNA, which is nonspecific for a particular gene, can thus result in many side effects (**Figure 4**). TSB are antisense oligonucleotides designed to bind perfectly to the region of the 3′-UTR complementary to the miRNA.

Recent developments on miRNAs have accelerated the evolution of methods and chemical modifications that make it possible to inhibit miRNAs in a stable manner, and to optimize their delivery. These are locked nucleic acids (LNA), peptide nucleic acids (PNA), phosphorothioate groups, miRNA sponges and nanoparticles (McKiernan et al., 2013; Nguyen and Chang, 2017). Phosphorothioate oligonucleotides increase the resistance to 3′-exonuclease hydrolysis and bind more promiscuously and with higher affinity to proteins than antisense oligonucleotides with phosphodiester linkages, which are present in natural DNA and RNA (Gilar et al., 1998; Crooke et al., 2017).

The base constituting the LNA is a nucleic acid analog in which the ribose ring is chemically modified with through the introduction of a methylene bridge. This chemical modification provides the molecule with great thermodynamic stability and prevents its deterioration by nucleases by reinforcing its affinity for its target (Lindow and Kauppinen, 2012).

miRNA sponges are constructs, or RNAs, which have several binding sites for a miRNA of interest, which makes it possible to limit its availability and therefore its action on associated targets (**Figure 4**). In melanoma, TYRP1 (tyrosinase-related protein 1) RNA acts like a sponge which, when associated with miR-16, limits its tumor suppressor activity. Inhibition of the targets with a binding blocker allows miR-16 to act on its target RAB17 and prevents cell proliferation (Gilot et al., 2017). This type of approach using the properties of miRNA sponges and LNA could be considered for therapeutic purposes.

The primary route of administration for oligonucleotides for systemic applications is by parenteral injection, including intravenous (IV) or subcutaneous injection (SCI). Following systemic administration, phosphorothioate-modified single-stranded ASOs rapidly transfer from the blood into tissues (minutes to hours). Following SC administration, ASOs are rapidly absorbed from the injection site into the circulation with peak plasma concentrations consistently reached within 3 to 4 h, followed by a much slower terminal elimination phase (half-life of up to several weeks). Cell uptake is predominantly mediated by endocytosis. The substitution of one non-bridging oxygen with the more hydrophobic sulfur atom, as phosphorothioate, increases both plasma stability and plasma protein binding and thus, ultimately, tissue bioavailability (Yu et al., 2013).

Pharmacokinetic properties of ASOs are similar across species and gender that facilitate drug development (Yu et al., 2007). In the case with all second-generation ASOs and for all animal species evaluated, ASOs distributes broadly into most tissues with the exception of the central nervous system after systemic administration. The major systemic tissues of distribution include liver, kidney, bone marrow, adipocytes (cell body but not lipid fraction), and lymph nodes (Geary et al., 2015). Once intracellular, ASOs exhibit long half-lives (2–4 weeks) and prolonged activity in suppressing or altering expression of their target RNA. Few data are available about the oral route of administration, because this strategy requires profound modification of the formulation for the delivery. An *in vivo* study have compared IV and oral administration of an ASO and demonstrated that the tissue distribution profile was similar following both routes of administration, with highest concentrations observed in the kidney followed by the liver, lymph nodes and spleen (Raoof et al., 2004). In the case of CF, inhaled ASOs could be considered because the lung remains the primary target and to dismissed side effects but we currently have no setback on this approach, which has only been carried out by two teams targeting chloride ion channels with modified activity in CF (Crosby et al., 2017; Sonneville et al., 2017).

The first clinical antagonist developed in clinical studies, miravirsen (SPC3649), is an antimiR-122, which targets and sequesters liver-specific miR-122. MiR-122 binds to the 5′-UTR of the hepatitis C virus (HCV) mRNA that it stabilizes, causing the virus to accumulate. Miravirsen has the advantage of being coupled to an LNA base (making it resistant to nucleases) and having phosphorothioate linkages. Miravirsen was initially tested in mice and African green monkeys (Elmen et al., 2008). Its action was then studied in chimpanzees chronically infected with HCV, and a prolonged decrease in viral replication, without any evidence of viral resistance or side effects, was observed in the treated animals. In 2011–2012, a phase IIa study was conducted in patients with chronic hepatitis C who received five SCIs per week of miravirsen for a period of 29 days. This clinical study showed a prolonged, dose-dependent decrease in viral RNA levels and cholesterol levels and a change in the expression of about 100 genes in the liver. Viral RNA levels became undetectable in five patients, with, however, a rebound in the virus’s expression in some of them (van der Ree et al., 2014). The variability of responses observed in patients suggests, however, that viral and/or host factors would influence response to treatment with miravirsen. Recently, a 5′-UTR C3U nucleotide change in HCV mRNA was demonstrated in patients who had a viral replication rebound (Ottosen et al., 2015). This mutant C3U is, in fact, insensitive to miravirsen. It would, therefore, make the virus independent of miR-122; the virus would then use an alternative mechanism to stabilize and replicate itself. The decrease in plasma levels of miR-122 in patients is still observed two months after the last injection of miravirsen, most likely due to the long half-life of miravirsen in tissues (about 30 days) (Matthes et al., 2016). Two pharmaceutical companies (*Roche* and *Regulus Therapeutics*) are currently involved in clinical trials to treat the HCV using antimiR-122.

In the case of CF, a team used TSBs which, by binding to the 3′-UTR of CFTR mRNA, prevents the binding of miR-101 and miR-145. The transfection of these molecules into the bronchial epithelial cells of patients with CF leads to an increase in CFTR expression and activity, suggesting that these TSBs could be used therapeutically (Viart et al., 2015). Other targets are being considered, like the protein ANO1 which, like CFTR, is involved in the secretion of chloride ions, pH regulation and the fluidity of airway surface liquid (Ruffin et al., 2013; Jun et al., 2016). It has shown that a TSB specifically preventing the binding of a miRNA (miR-9) on the 3′-UTR of the mRNA of the alternative chloride channel ANO1, made it possible, in *in vitro* and *in vivo* models, to increase its expression, and also its chloride activity, and cell migration, as well as mucociliary clearance independently of intracellular calcium concentration (Sonneville et al., 2017). The restoration of these parameters, deregulated at a physiological level, makes it possible to now propose the TSB ANO1 as a potential therapeutic target in the context of CF. Each patient would be able to benefit from this type of approach leading to the activation of a chloride secretion independent of CFTR. In this study, the TSB ANO1 was administered to animals intranasally, if this molecule were to be used in humans, its mode of administration would need to be determined. Inhalation seems to be the most appropriate method since these patients often have lung damage. Tissue-specific, it has less systemic exposure, reducing the risk of side effects. However, the patients’ lungs have barriers, such as airways that are obstructed by mucus, that can make administration difficult. The passage of TSBs through the mucus therefore still needs to be studied.

## Conclusion

Despite the improved care and recent progress in the identification of new therapies the predicted median survival of CF patients still remains in the low 40-years range in developed countries. New therapies and new strategies, alone or in combination with established therapies, are needed to prolong survival and improve the quality of life for all CF patients. The potential for modulating gene expression by the use of antisense oligonucleotides has become increasingly interesting in recent years, but safe delivery, long-term efficacy and side effects of prolonged treatment still need to be assessed. Advances in chemistry and molecular biology have provided the basis to develop antisense oligodeoxynucleotides and improve their selectivity, stability, and specificity of action. The antisense oligonucleotide drug platform is a really new approach for drug discovery, but basic science must be improved notably in understanding the molecular mechanisms that regulate CFTR and other chloride channels. Therapies targeting CFTR increase with ASO should be limited because channel increase will not necessarily induce increased activity especially for class III to VI mutations. The ease of delivery of modified antisense oligonucleotides seems to be linked with a lack of any major adverse side effects, making antisense oligonucleotides suitable candidates as a potential treatment for CF diseases.

## Author Contributions

PB, FS, and OT wrote the manuscript. PB, FS, HC, and OT participated in the design of this manuscript.

## Conflict of Interest Statement

The authors declare that the research was conducted in the absence of any commercial or financial relationships that could be construed as a potential conflict of interest.

## Footnotes

1 www.genet.sickkids.on.ca/cftr

2 https://www.cff.org/Trials/Pipeline
